# Proline dehydrogenase from *Thermus thermophilus* does not discriminate between FAD and FMN as cofactor

**DOI:** 10.1038/srep43880

**Published:** 2017-03-03

**Authors:** Mieke M. E. Huijbers, Marta Martínez-Júlvez, Adrie H. Westphal, Estela Delgado-Arciniega, Milagros Medina, Willem J. H. van Berkel

**Affiliations:** 1 Laboratory of Biochemistry, Wageningen University & Research, Stippeneng 4, 6708 WE Wageningen, The Netherlands; 2 Department of Biochemistry and Molecular Cell Biology and Institute for Biocomputation and Physics of Complex Systems, University of Zaragoza, Pedro Cerbuna 12, 50009, Zaragoza, Spain

## Abstract

Flavoenzymes are versatile biocatalysts containing either FAD or FMN as cofactor. FAD often binds to a Rossmann fold, while FMN prefers a TIM-barrel or flavodoxin-like fold. Proline dehydrogenase is denoted as an exception: it possesses a TIM barrel-like fold while binding FAD. Using a riboflavin auxotrophic *Escherichia coli* strain and maltose-binding protein as solubility tag, we produced the apoprotein of *Thermus thermophilus* ProDH (MBP-TtProDH). Remarkably, reconstitution with FAD or FMN revealed that MBP-TtProDH has no preference for either of the two prosthetic groups. Kinetic parameters of both holo forms are similar, as are the dissociation constants for FAD and FMN release. Furthermore, we show that the holo form of MBP-TtProDH, as produced in *E. coli* TOP10 cells, contains about three times more FMN than FAD. In line with this flavin content, the crystal structure of TtProDH variant ΔABC, which lacks helices αA, αB and αC, shows no electron density for an AMP moiety of the cofactor. To the best of our knowledge, this is the first example of a flavoenzyme that does not discriminate between FAD and FMN as cofactor. Therefore, classification of TtProDH as an FAD-binding enzyme should be reconsidered.

Flavoenzymes are ubiquitous in nature and function as versatile biocatalysts. They play an essential role in various biological processes such as biosynthesis, energy production, light emission, biodegradation, chromatin remodelling, DNA repair, apoptosis, protein folding, detoxification, and neural development^1^.

Flavoenzymes usually contain flavin mononucleotide (FMN) or flavin adenine dinucleotide (FAD) as redox active prosthetic group (Fig. 1). These cofactors are synthesised from riboflavin (vitamin B2) through the action of riboflavin kinase (E.C. 2.7.1.26) and FMN adenylyltransferase (E.C. 2.7.7.2), respectively. Eukaryotes and some archaea depend on two seperate enzymes for FMN and FAD synthesis^2^,^3^, but in most prokaryotes these two enzymes are fused into a bifunctional protein, FAD synthetase^4^,^5^,^6^. FAD is utilised three times more often than FMN as enzyme prosthetic group, while riboflavin is not used for this purpose^7^,^8^.

FMN- and FAD-dependent enzymes have a preference for certain protein folds. While FMN enzymes show a preference for a TIM-barrel or flavodoxin-like fold, FAD enzymes often use a Rossmann fold for binding the ADP dinucleotide moiety of the cofactor^7^. Some flavoenzymes, like NADPH cytochrome P450 oxidoreductase and nitric oxide synthase, use both flavin cofactors for the transport of electrons through separate domains^9^,^10^.

The majority (90%) of flavoproteins bind their cofactor non-covalently^7^. Flavin dissociation provides the opportunity of studying the properties of the apoenzyme^11^,^12^, and allows for reconstituting the holoprotein with isotopically enriched^13^ or artificial^14^ flavins. Furthermore, apoflavoenzymes can be selectively immobilised by anchoring to a flavin-containing carrier^15^. Different methods have been explored to dissociate flavoproteins into apoprotein and flavin prosthetic group^16^. Traditional methods include precipitation procedures and dialysis^17^. However, these approaches may give low yields, since many apoflavoproteins cannot withstand the harsh and/or time-consuming conditions employed. More recent methods for apoprotein preparation focus on reversible immobilisation strategies, which allow deflavination and reconstitution of preparative amounts of flavoprotein^18^,^19^. Particularly for flavoproteins with a more complex quaternary structure, production of fully reconstitutable apoprotein remains challenging.

In some flavoproteins the flavin is covalently bound to the polypeptide chain^20^,^21^. By replacing the target residue(s) of covalent flavinylation through site-directed mutagenesis, the apoprotein can be obtained^22^,^23^. For vanillyl-alcohol oxidase it could be established that the FAD becomes covalently linked to the protein in an autocatalytic process, and that the initial non-covalent binding of FAD to the apo dimer stimulates enzyme octamerisation^24^,^25^.

An alternative approach for the preparation of apoenzyme is the use of riboflavin-deficient expression systems. The *E. coli* strain BSV11 is riboflavin auxotrophic^26^. It carries a mutation in the ribB gene, which encodes for 3,4-dihydroxy-2-butanone-4-phosphate synthase, an essential enzyme in the riboflavin biosynthesis pathway^27^. Using riboflavin auxotrophic strains, both flavoenzymes that bind their flavin covalently or non-covalently can be produced in their apo-form. Examples include sarcosine oxidase^28^ and vanillyl-alcohol oxidase^29^.

Proline dehydrogenase (ProDH, EC 1.5.5.2) is a ubiquitous flavoenzyme involved in proline catabolism^30^,^31^. It oxidises L-proline to Δ^1^-pyrroline-5-carboxylate (P5C), which is non-enzymatically hydrolysed to glutamic semialdehyde (GSA). P5C dehydrogenase (P5CDH, EC 1.2.1.88) oxidises GSA to L-glutamate. ProDH and P5CDH exist as monofunctional enzymes in some bacteria and in eukaryotes; however, in other bacteria they are fused into a bifunctional enzyme called proline utilization A (PutA)^32^,^33^.

In humans, malfunctioning of the proline metabolic enzymes can lead to several medical issues^34^,^35^. The gene encoding for human ProDH (also known as proline oxidase) is a hot-spot for mutations^36^. Missense mutations in ProDH have been identified in patients suffering from hyperprolinemia and the neuropsychiatric disorder schizophrenia^37^,^38^,^39^,^40^. Furthermore, ProDH is one of the genes markedly induced by tumour suppressor p53^41^,^42^ and plays a role in tumorigenesis and tumour development^43^.

ProDH adopts a distorted (βα)_8_ TIM-barrel fold^32^,^33^,^44^. Next to methylenetetrahydrofolate reductase (MTHFR)^45^, ProDH is the only known TIM-barrel enzyme that contains an FAD cofactor. Due to this property, ProDH and MTHFR have been structurally classified as separate clans of FAD oxidoreductases^7^.

Previously, we described the properties of *Thermus thermophilus* ProDH (TtProDH), produced through fusion with maltose-binding protein (MBP)^46^. Because MBP-TtProDH appeared to be prone to aggregation, we constructed a variant with a more polar N-terminus (F10E/L12E). This MBP-TtProDH variant, here further referred to as EE, forms homogeneous tetramers^47^. Each TtProDH subunit binds a FAD cofactor^32^. However, molecular details of FAD incorporation are currently unknown.

In this communication, we describe the properties of the EE apoprotein. Using a riboflavin auxotrophic expression strain, properly folded apoenzyme was obtained. Intriguingly, we discovered that EE accepts both FAD and FMN as prosthetic group. Based on this finding, the flavin composition and mode of cofactor binding of TtProDH was further investigated.

## Results

### Preparation of apo-EE

Initial attempts of producing reconstitutable apoprotein from purified holo-EE were not successful. When using precipitation, dialysis or affinity chromatography-based deflavinylation protocols^16^, irreversible aggregation of the apoenzyme occurred. Therefore, we focused our attention on producing apo-EE in a cellular environment. Using the riboflavin auxotrophic *E. coli* strain BSV11 as expression host, the apoenzyme was properly produced with purification yields of approximately 10 mg/L culture. The preparation contained mainly apo-EE and a minor amount of holo-EE, as judged from the low flavin absorbance in the visible region and the low activity compared with native enzyme (*vide infra*).

### Reconstitution of apo-EE

**Figure 2:**
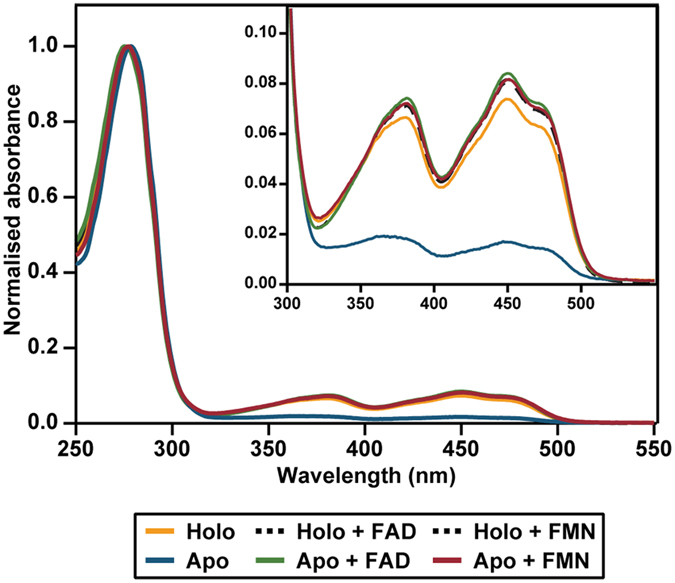
Absorption spectra of holo-EE, apo-EE and apo-EE reconstituted with FAD or FMN.

Apo-EE shows a low flavin absorption in the visible region, yielding an A_280_/A_450_ ratio of 58.9 (Fig. 2). The apoenzyme can be successfully reconstituted with FAD or FMN, yielding absorption spectra similar to that of holo-EE (Fig. 2). The A_280_/A_450_ ratios for apoenzyme reconstituted with FAD (11.4) and FMN (12.0) are similar to that of holo-EE, pre-incubated with excess FAD (11.8) or FMN (12.1). Holo-EE, as purified here in the absence of excess FAD, shows an A_280_/A_450_ ratio of 13.3. This indicates that holo-EE contains a small amount of apoprotein.

**Figure 3:**
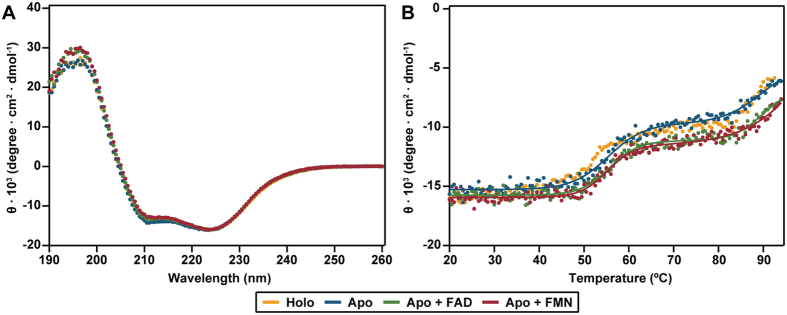
Secondary structure and thermal stability of holo-, apo-, and reconstituted EE. (**A**) Far-UV CD spectra. (**B**) Thermal unfolding monitored by CD at 224 nm.

Far-UV CD spectra of holo-EE, apo-EE and reconstituted apo-EE are all very similar, indicating no large changes in secondary structure (Fig. 3A). Thermal unfolding monitored by CD spectroscopy shows two separate transitions for each sample (Fig. 3B). This indicates non-cooperative unfolding for the MBP and TtProDH domains^46^. The first transition reflects unfolding of MBP, with a midpoint of unfolding around 55 °C. The thermostable TtProDH domain has a midpoint of unfolding around 90 °C. When comparing the thermal stability of apo-EE to that of reconstituted apo-EE or holo-EE, no large differences are observed.

Results from native mass spectrometry have shown that holo-EE forms tetramers with a mass of 319.6 ± 33 kDa^47^. Apo-EE is also a tetramer, with a native mass of 310.7 ± 31 kDa. Therefore, the oligomerisation state of the enzyme is not affected in the absence of the flavin cofactor.

### Catalytic properties of reconstituted apo-EE

**Figure 4:**
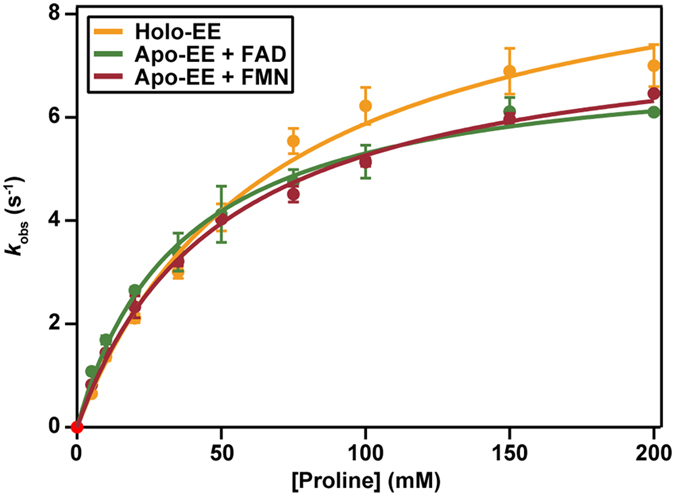
Steady-state kinetics of holo-EE and apo-EE reconstituted with FAD and FMN, as determined with the proline: DCPIP assay.

Comparison of the specific activities of apo-EE and holo-EE shows that the obtained apoprotein preparation has about 17% residual activity (Table 1). Apo-EE reconstituted with FAD or FMN shows equal specific activities as holo-EE, indicating that the apoenzyme can be fully reconstituted with either of the flavin cofactors. The kinetic parameters of apo-EE, reconstituted with FAD or FMN, are very similar to those described for holo-EE (Table 1 and Fig. 4), indicating no preference for either of the prosthetic groups in catalysis.

### Cofactor binding of apo-EE

**Figure 5:**
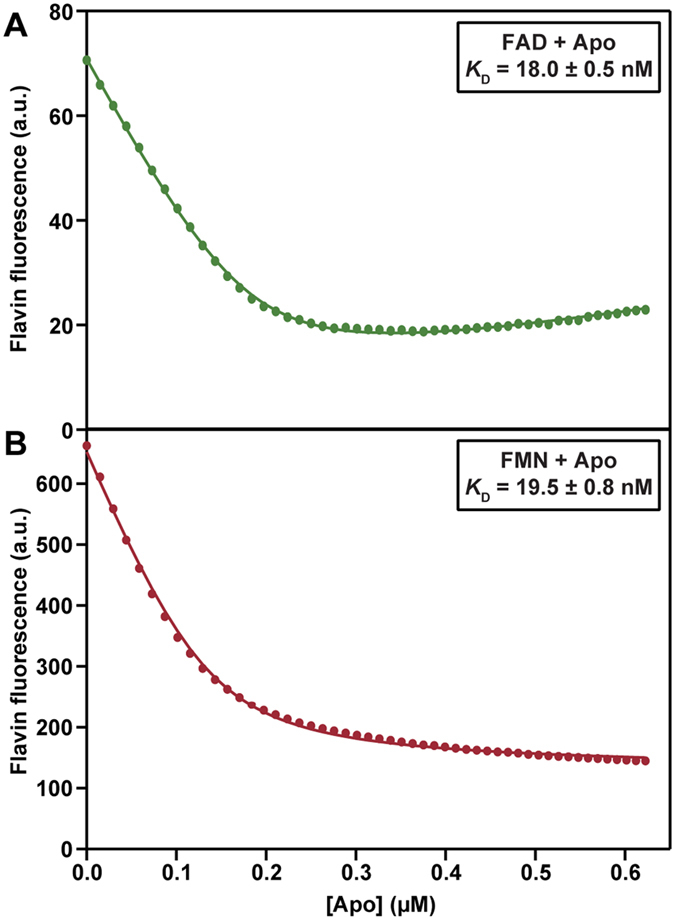
Titration of an FAD or FMN solution with apo-EE to determine the dissociation constants of the apoenzyme-FAD/FMN complex. Flavin fluorescence emission was monitored at 525 nm, excitation was at 445 nm. (**A**) 200 nM FAD titrated with 5 μM of apo EE and (**B**) 200 nM FMN titrated with 5 μM apo EE.

The equilibrium constants for dissociation of FAD and FMN from EE were determined using fluorescence spectroscopy. Upon binding to the apoprotein, fluorescence of the flavin is severely quenched within seconds. From titrating aliquots of apoenzyme to an FAD or FMN containing solution (Fig. 5), tight binding is observed and equal dissociation constants for the apo-FAD (*K*_D_ = 18.0 ± 0.5 nM) and apo-FMN (*K*_D_ = 19.5 ± 0.8 nM) complexes are estimated. Of note is that riboflavin shows a very weak binding to the apoenzyme (data not shown). From Fig. 5 it is evident that the fluorescence quantum yield of the apo-FAD complex is lower than the fluorescence quantum yield of the apo-FMN complex. The increase in flavin fluorescence at the end of the FAD titration (Fig. 5A) can be attributed to the residual amount of flavin present in the apoprotein preparation.

**Figure 6:**
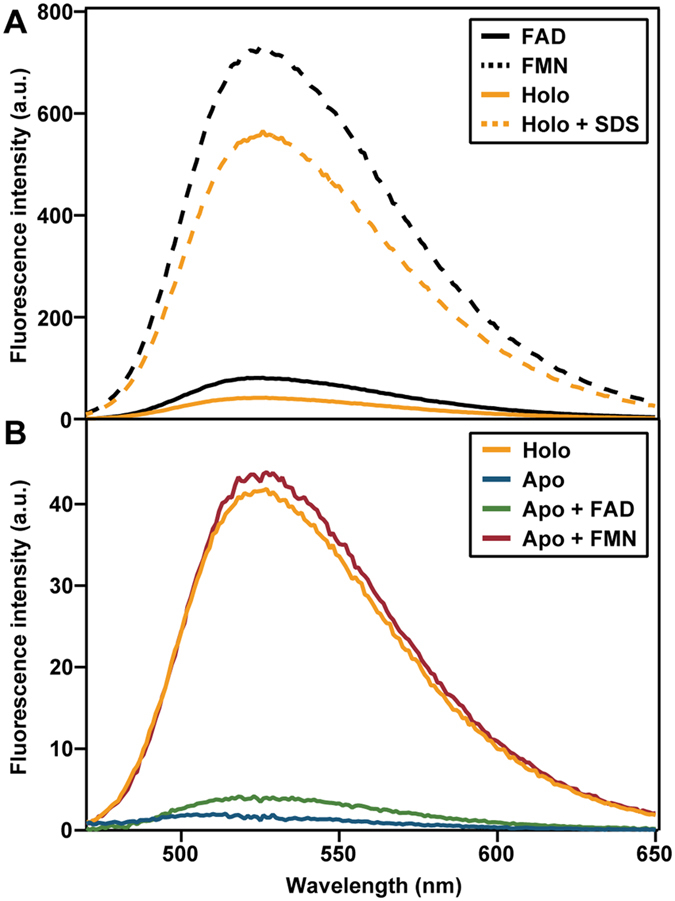
Fluorescence emission spectra of of holo- and apo-EE. Excitation was at 445 nm. (**A**) Spectra of 0.9 μM FAD, 0.9 μM FMN, 0.9 μM holo-EE and 0.9 μM holo-EE denatured in 0.5% SDS. (**B**) Spectra of 0.9 μM apo-EE and 0.9 μM apo-EE reconstituted with FAD or FMN. For comparison, the spectrum of holo-EE is depicted.

FAD in solution is about 9 times less fluorescent than FMN (Fig. 6A). This is due to stacking of the adenine part onto the isoalloxazine ring of FAD, which quenches its fluorescence^48^,^49^. In TtProDH, FAD binds in an extended conformation, as deduced from the crystal structure^32^. Nevertheless, binding to the apoprotein severely quenches the fluorescence of FAD (Figs 5A and 6B). This is also true for FMN, but the fluorescence quantum yield of the apoenzyme reconstituted with FMN is significantly higher than the fluorescence quantum yield of the apoenzyme reconstituted with FAD (Figs 5B and 6B).

The emission spectrum of holo-EE, isolated from *E. coli*, shows much higher fluorescence intensity than that of the reconstituted apo-FAD complex (Fig. 6B). This suggests that next to FAD, holo-EE might also contain FMN. By denaturing holo-EE with 0.5% SDS (Fig. 6A), the flavin cofactor composition could be measured more accurately. From fluorescence calibration curves of FMN and FAD, it was estimated that 75 ± 3% of the released flavin is FMN. To confirm that the enzyme incubated with 0.5% SDS had released all its cofactor, part of the sample was passed through a 10 kDa cut off spin filter to remove the enzyme before measuring fluorescence emission spectra and part of the sample was measured directly. The obtained spectra were identical, indicating all enzyme had released its cofactor and only the fluorescence of flavin free in solution was recorded. As mentioned, no extra flavin was added during purification of holo-EE, therefore, the FAD/FMN ratio was not altered by experimental procedures.

The cofactor composition of holo-EE purified from *E. coli* TOP10 cells was also determined using mass spectrometry. The flavin cofactor was extracted from the enzyme using 60% ethanol. FAD was detected in positive mode for the transition of 786.15 to 136.10 m/z and from 786.15 to 348.10 m/z at 8.85 min (Fig. S1). FMN was detected in negative mode for the transition of 455.00 to 97.00 m/z and from 455.00 to 78.90 m/z at 8.73 min (Fig. S1). From the measured transitions for the purified flavin cofactor the concentrations for FMN and FAD can be calculated, which indicates that about 74% of the flavin cofactor in holo-EE is FMN and only 26% is FAD, matching perfectly with the fluorescence emission properties. This FAD/FMN ratio in holoenzyme might depend on the cellular conditions, and therefore this ratio might differ per protein batch.

### Crystal structure of TtProDH ΔABC

**Figure 7:**
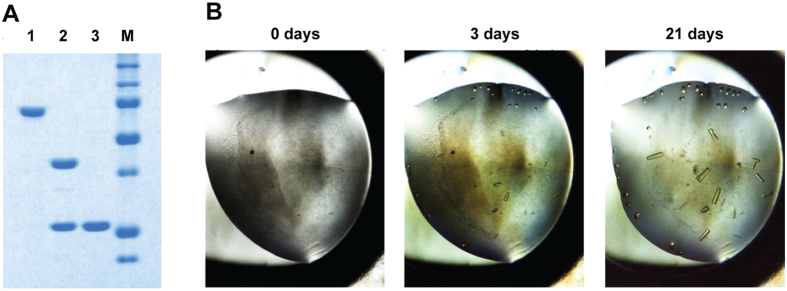
Preparation of ΔABC for crystallisation. (**A**) Removal of the MBP-tag and purification of native ΔABC, visualised on SDS-PAGE. From left to right: (1) purified MBP-TtProDH ΔABC; (2) MBP-TtProDH ΔABC after limited trypsinolysis; (3) purified native ΔABC. Molecular masses of marker proteins (M) from top to bottom: 150, 100, 75, 50, 37, 25, 20 kDa. (**B**) Growth of TtProDH ΔABC crystals.

Crystallisation of EE was hampered by inconvenient removal of the MBP tag^47^. We created a TtProDH variant (ΔABC), which lacks the N-terminal helices αA, αB and αC. ΔABC is poorly active but binds the flavin cofactor in stoichiometric amounts. From this variant, the MBP-tag could be successfully removed (Fig. 7A) and the purified ProDH fragment was used for crystallisation. Crystals of TtProDH ΔABC reached their maximum size in 21 days (Fig. 7B). Collection statistics and refinement data of the crystals obtained are summarised in Table 2. The coordinates and structure factors have been deposited in the Protein Data Bank (PDB) with accession code 5M42.

**Figure 8:**
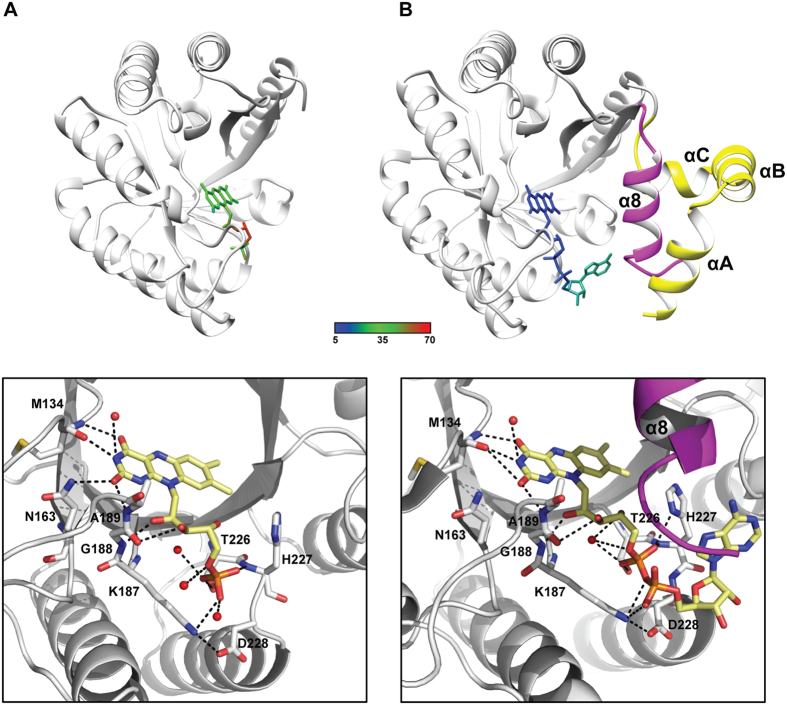
Cartoon representation of the three-dimensional models of the crystal structures of (**A**) TtProDH ΔABC and (**B**) TtProDH (PDB entry 2G37). In the top panels, the flavin cofactors are coloured by B-factor. N-terminal helices αA, αB, and αC and the C-terminal α8 are coloured in yellow and magenta, respectively, in TtProDH and are missing in TtProDH ΔABC. The bottom panels show the H-bond network contributing to stabilisation of the cofactor. Involved residues are labelled and represented as CPK sticks and flavin cofactors show their Cα in pale yellow. Water molecules are depicted as red spheres.

**Figure 9:**
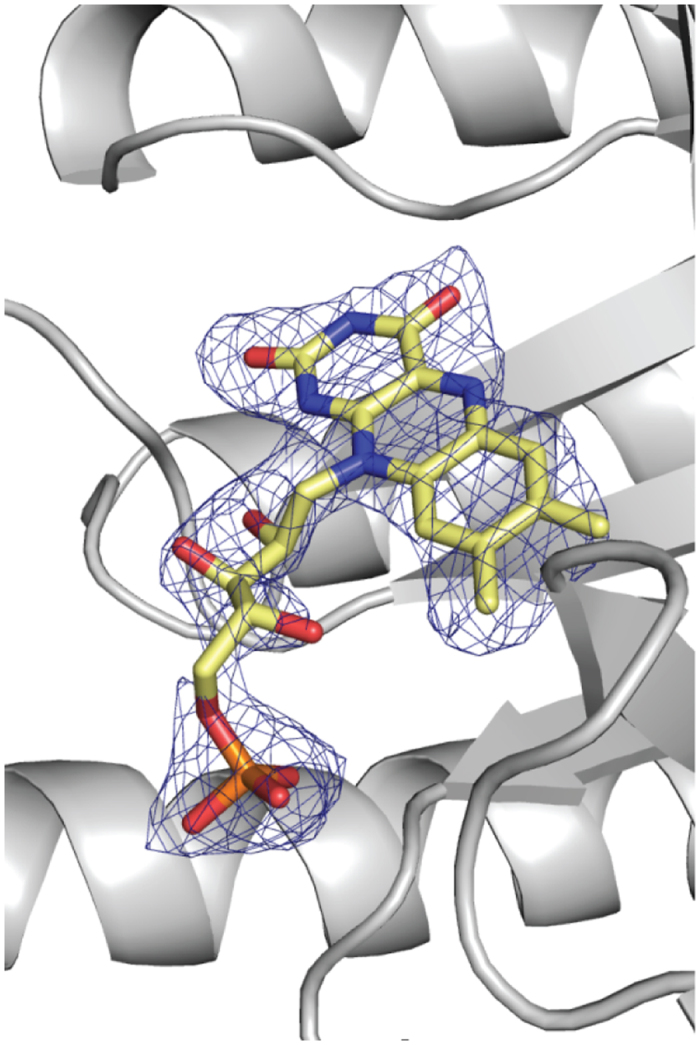
Fo-Fc omit map (contoured at 2.2 σ, in blue) for the bound flavin cofactor of TtProDH ΔABC. FMN is shown in CPK sticks with carbons in yellow.

The three-dimensional model for TtProDH ΔABC comprises residues 38–279 (Fig. 8A). Superposition of this structure onto that of TtProDH (2G37) showed an rmsd value of 0.338 Å (for 221 Cα atoms of A chains) demonstrating a similar overall structure and no gross conformational changes. Using ESI-MS, we detected that the C-terminal helix α8 of MBP-TtProDH ΔABC is unstable and becomes proteolytically cleaved in *E. coli* after Thr287. While MBP-TtProDH has a predicted denatured mass of 74401 Da, the truncated form has a measured mass of 71899 Da. This points to removal of a part of the C-terminal tail with sequence (288-RRIAERPENLLLVLRSLVSGLE-309). This truncated form has a predicted subunit mass of 71885 Da. Deletion of the last 22 residues might increase the flexibility of the remaining C-terminal end of the protein and explain why residues 280–287 are not visible in the crystal structure. For the FAD cofactor, no electron density was observed for the adenosine 5′-monophosphate (AMP) moiety (Fig. 9). This suggests that either the AMP part of the FAD shows multiple orientations and therefore is highly mobile, or that (part of) the bound cofactor is FMN instead of FAD. The mode of binding of the FMN-part of the flavin cofactor is strictly conserved compared to that of TtProDH (Fig. 8B).

To confirm that ΔABC binds both FMN and FAD, the flavin content of TtProDH ΔABC was determined using fluorescence and mass spectroscopy, as described above for holo-EE. This revealed that TtProDH ΔABC contains 78 ± 5% FMN and 22 ± 5% FAD, similar as for holo-EE.

## Discussion

In this manuscript, we describe for the first time the production of fully reconstitutable apoprotein of proline dehydrogenase. Using the riboflavin auxotrophic *E. coli* strain BSV11, we obtained an apoprotein preparation that contained 17% residual activity. Spectral analysis confirmed the presence of some holoenzyme, analogous to observations made with sarcosine oxidase and vanillyl-alcohol oxidase^28^,^29^. More striking, the obtained apoenzyme can be fully reconstituted with either FMN or FAD, leading to quite identical catalytic properties.

ProDH and MTHFR are the only FAD-binding enzymes with a (βα)_8_ TIM-barrel fold^7^. The presence of a non-covalent FAD cofactor in ProDH was first described by Scarpulla and Soffer in 1978^50^. They showed that the activity of ProDH, which was solubilised from *E. coli* membranes, is stimulated by FAD but not FMN. After that, it was shown that the flavin cofactor of PutA from *Salmonella typhimurium*, assumed to be FAD, could be reduced by proline^51^,^52^. Since these observations, ProDH has been denoted as an FAD-containing enzyme. We show here that TtProDH does not limit itself to FAD as cofactor; it also binds FMN with equal affinity and has similar kinetic parameters with both cofactors. Moreover, heterologously overproduced MBP-TtProDH contains more FMN than FAD. The fact that apo-EE and holo-EE have similar spectral and hydrodynamic properties suggests that apo-EE is fully folded and awaits flavin binding. This is similar as in e.g. flavodoxin^53^, *para*-hydroxybenzoate hydroxylase^12^ and VAO^24^. However, in VAO, initial non-covalent binding of FAD to the apo dimer stimulates enzyme octamerisation and autocatalytic flavinylation^25^.

Up to now, all known crystal structures of PutAs and ProDHs contain an FAD cofactor^32^,^44^,^54^,^55^,^56^. In the crystal structure of TtProDH (PDB entry 2G37), the high B-factor around the adenosine part of the cofactor is due to the fact that the adenosine is not in contact with any atoms or water molecules in the structure (Fig. 8B). This supports an increased flexibility or absence of the adenosine moiety in a fraction of the enzyme.

The PutAs and ProDHs that have been analysed so far have a similar location for the isoalloxazine ring system and diphosphoribose moiety of the FAD cofactor, but the orientation of the adenosine group differs between the two enzyme groups. This diverse orientation might be caused by the structural differences between PutAs and ProDHs. First, PutAs contain an additional helix, α5a, which is replaced by a loop in monofunctional ProDHs. Helix α5a contains a tryptophan that stacks against the adenine group of the FAD in PutAs. The loop in ProDHs does not have an equivalent of this tryptophan for interaction with the FAD. Second, PutAs have extra helical stretches that follow after helix α8. These additional C-terminal helices in PutAs would clash with the conformation of the adenine ring of the FAD cofactor as it is found in monofunctional ProDHs^32^,^57^.

Often, the ADP moiety strongly contributes to the interaction between FAD and the apoprotein^24^,^58^,^59^,^60^. However, in TtProDH, the adenosine moiety of FAD does not show any interaction with the enzyme^32^ (Fig. 8B). The flavin cofactor inserts its ribityl pyrophosphate moiety next to strands 5 and 6. There are several interactions between TtProDH and the pyrophosphate of FAD. Thr226 and His227, present in the β6-α6 loop, interact with the FMN phosphate. Lys187, stabilised by Asp228, contacts the AMP phosphate. These residues are all present in conserved sequence motifs of the PutA/ProDH family, with His227 and Lys187 being strictly conserved throughout the family^32^.

Flavoenzymes that adopt TIM-barrel folds and bind FMN, such as glycolate oxidase^61^, flavocytochrome *b*_2_^62^, old yellow enzyme^63^, trimethylamine dehydrogenase^64^, and dihydroorotate dehydrogenase^65^, insert the ribityl phosphate moiety next to strands 7 and 8. With these enzymes, the phosphate group interacts with amides in the initial turn of the short helix α8’. In MTHFR^45^, the only other known TIM-barrel enzyme that binds FAD, the ribityl chain extends between barrel strands 4 and 5, and the FMN phosphate binds to the β4-α4 loop. In addition, the adenosine moiety of the FAD cofactor in MTHFR does show several contacts with the enzyme. These data suggest that TtProDH has a different binding mode for the FAD/FMN cofactor compared to other FMN-binding TIM-barrel enzymes, and to the FAD-binding MTHFR.

The TtProDH variant ΔABC reveals that helices αA, αB, αC and α8 are not essential for binding of the flavin cofactor in TtProDH. Based on these observations and the present results, we conclude that there is no structural reason why TtProDH should not bind FMN. Thus, we suggest that PutAs might be specific for FAD, whereas monofunctional ProDHs do not necessarily discriminate between FAD and FMN as cofactor.

When purified from *E. coli*, the majority of the EE variant of MBP-TtProDH contains FMN as prosthetic group. This might be explained by the availability of FMN and FAD in the cell. The ability of TtProDH to bind both FAD and FMN makes the enzyme less dependent on the availability of both cofactors in the cell. Next to TtProDH, NADH oxidase from *Thermus thermophilus* has been shown to function with both FAD and FMN^66^. However, with the NADH oxidase the dissociation constants for both cofactors differ by a few orders of magnitude. This suggests that this enzyme tolerates FAD as cofactor, while FMN is its natural cofactor^67^. To the best of our knowledge, TtProDH is the first example of a flavoenzyme that does not discriminate between FAD and FMN as cofactor. Therefore, classification of TtProDH as an FAD-binding enzyme should be revised.

## Materials and Methods

### Enzyme production and purification

*E. coli* TOP10 cells containing a pBAD plasmid (pBAD-EE), in which the changes F10E/L12E were introduced in the gene encoding MBP-TtProDH^47^, were used for heterologous expression of holo MBP-TtProDH EE. The holo EE was purified as described previously^47^, except that no extra FAD was added during purification. Riboflavin auxotrophic *E. coli* BSV11 strain was used as expression host for production of apo-EE. This strain is defective in riboflavin synthesis and was obtained by Tn5 transposon mutagenesis^13^. For enzyme production, a 10 mL pre-culture inoculated with cells harbouring the pBAD-EE plasmid was grown o/n at 37 °C in Luria-Bertani (LB) medium containing 100 μg/mL ampicillin and 50 μM riboflavin. The pre-culture was used to inoculate two 2 L Erlenmeyer flasks, each containing 500 mL LB medium, 100 μg mL^−1^ ampicillin and 50 μM riboflavin. The cells were grown at 37 °C until OD_600_ = 0.8. Cells were spun down (7000 g for 10 min) and washed three times with riboflavin-free LB. After the third washing step, the cells were divided over two 2 L erlenmeyer flasks, both containing 500 mL riboflavin-free LB medium and 100 μg/mL ampicillin. After one hour shaking (200 rpm) at 20 °C, protein expression was induced by adding 0.02% (w/v) L-arabinose, and growth and expression continued for 14 h. Cells (6 g, wet weight) were harvested by centrifugation (7000 g for 10 min at 4 °C) and resuspended in 50 mM sodium phosphate, pH 7.4. After addition of 1 mg DNaseI, 1 mM MgCl_2_ and a Complete Protease Inhibitor Cocktail Tablet (Roche Diagnostics), cells were lysed by passing them three times through a pre-cooled French Press pressure cell (SLM Amicon Instruments) at 10000 psi. Cell lysate was centrifuged at 26000 g for 1 h at 4 °C. Apo-EE was purified using an amylose column (New England Biolabs, 20 mL in XK 16/10), and a Source 15Q column (GE Healthcare, 20 mL in XK 16/10), according to a protocol that has been described before for the holoenzyme^46^,^47^. In addition, after the ion exchange column, the enzyme was concentrated using a 10 kDa cut off Amicon filter and loaded on a preparative Superdex200 XK26/1000 column (GE Healthcare), equilibrated in 50 mM sodium phosphate, 150 mM NaCl, pH 7.4. The eluted protein was concentrated and simultaneously the buffer was changed to 50 mM sodium phosphate, pH 7.4 by using a 30 kDa cutoff Vivaspin 6 spinfilter (GE Healthcare). Protein concentrations were determined using the bicinchoninic acid (BCA) assay (Thermo Scientific) and the purified apoenzyme with a concentration of 7.5 mg/mL was flash-frozen in liquid nitrogen and stored at −80 °C. The native mass of apo-EE was determined using nanoflow electrospray ionisation mass spectrometry (ESI-MS), as described before^46^,^47^.

A helical arm-truncated variant lacking N-terminal helices αA, αB and αC was constructed. The plasmid pBAD-MBP-TtProDH^47^ was PCR-amplified using the primers 5′AAT TAG AAT TCA TGG CGA AAA TTG AAA CCC TGG AAG AAG CAC TG 3′ (forward) and 5′ GCC CAA GCT TTT ATT CTA GAC CGC TAA CCA GGC 3′ (reverse). Using *Eco*RI and *Hind*III restriction sites (underlined in the primers), the amplified fragment was introduced into a pBAD-MBP vector, which resulted in an N-terminal fusion of the TtProDH ΔABC variant to MBP. The resulting construct was verified by automated sequencing of both strands (Macrogen). *E. coli* TOP10 cells were transformed with the plasmid for recombinant expression. MBP-TtProDH ΔABC was produced and purified as described previously^47^. The denatured mass of MBP-TtProDH ΔABC was determined using nanoflow electrospray ionisation mass spectrometry (ESI-MS)^46^. Removal of the MBP-tag and purification of native ΔABC was achieved by slight modification of the trypsin-treatment reported before^46^. In this case, 25 mg purified MBP-TtProDH ΔABC in 50 mM sodium phosphate, pH 7.4 was incubated with 36 μg trypsin at 37 °C for 1 h. After that, phenylmethylsulfonyl fluoride (PMSF, Merck) was added to a final concentration of 2 mM to inactivate trypsin and the protocol to purify ΔABC was proceeded as described^46^. Trypsinolysis of MBP-TtProDH ΔABC and purification of ΔABC was visualised with sodium dodecyl sulphate polyacrylamide gel electrophoresis (SDS-PAGE), using 12% polyacrylamide slab gels. Proteins were stained using Coomassie Brilliant Blue R-250. As a molecular weight marker, Precision Plus Protein Standard (Biorad) was used.

### Spectral analysis

Holo-EE (10 μM) and apo-EE (10 μM) were incubated with 50 μM FAD or FMN, in 50 mM sodium phosphate, pH 7.4 at room temperature for 1 hour. Excess FAD/FMN was removed using a 10 kDa cut off spin filter and flavin absorption spectra of holo-EE, apo-EE and reconstituted enzymes were recorded at 25 °C on a Hewlett-Packard 8453 diode array spectrophotometer, essentially as described before^46^.

Far-UV circular dichroism (CD) spectra of holo-EE, apo-EE and apo-EE reconstituted with FAD or FMN were acquired on a Jasco J-715 spectropolarimeter equipped with a Peltier thermostat (Jasco). Samples contained 1 μM enzyme in 50 sodium phosphate, pH 7.4 and spectra were recorded as described previously^46^. Temperature-induced unfolding was monitored by increasing the temperature from 20–95 °C at a rate of 0.5 °C min^−1^. Data points were collected every 0.5 °C increase. Midpoints of transition were determined by fitting the data to a model described by a double sigmoidal function.

### Enzyme activity

Enzyme activity of apo-EE, apo-EE reconstituted with FAD or FMN, and holo-EE was determined at 25 °C on a Hewlett Packard 8453 diode array spectrophotometer using the proline:dichlorophenolindophenol (DCPIP) oxidoreductase assay^46^. For the standard assay, catalytic amounts of enzyme were added to a 600 μL reaction mixture containing 65 μM DCPIP and 100 mM L-proline in 50 mM sodium phosphate, pH 7.4. Steady-state kinetic parameters were determined at 25 °C, essentially as described previously^47^.

### Fluorescence spectroscopy

Dissociation constants of apo-EE-flavin complexes were determined by using the quenching of flavin fluorescence upon binding of the flavin cofactor to the apoenzyme. 200 nM solutions of FAD, FMN or riboflavin in 50 mM sodium phosphate, pH 7.4, were prepared based on the molar absorption coefficients of 11300 M^−1^ cm^−1^ at 450 nm for free FAD, 12200 M^−1^ cm^−1^ at 445 nm for free FMN and 12500 M^−1^ cm^−1^ at 445 nm for free riboflavin. 1.3 mL of the 200 nM FAD, FMN or riboflavin solutions were titrated with 5 μL aliquots of 5 μM apoenzyme in the same buffer. In total, 250 μL of the enzyme solution was added. After addition of each aliquot of enzyme, fluorescence emission was recorded during 30 sec. Excitation was at 445 nm (bandwidth 5 nm) and emission at 525 nm (bandwidth 10 nm) and the photomultiplier potential was set at 975 V. As control, buffer (50 mM sodium phosphate pH 7.4) was titrated with apoenzyme under the same conditions.

The dissociation constants (*K*_D_) of the apo-EE-FAD/FMN complexes were determined using Igor Pro 6.10, by fitting the fluorescence emission data (*F*_*total*_) to the model described by eq. 1:


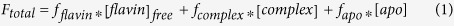


where


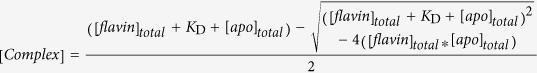


and [*flavin*]_*total*_ and [*apo*]_*total*_ are the concentrations of flavin and apoenzyme at each point in the titrations. *f*_*flavin*_, *f*_*complex*_ and *f*_*apo*_ are the fluorescence conversion factors for each species, respectively.

Fluorescence emission spectra of 0.9 μM FAD, 0.9 μM FMN, 0.9 μM apo-EE reconstituted with FAD, and 0.9 μM apo-EE reconstituted with FMN were recorded. All solutions were prepared in 50 mM sodium phosphate, pH 7.4. Excitation was at 445 nm (bandwidth 5 nm) and emission at 470–650 nm (bandwidth 10 nm). The photomultiplier potential was set at 820 V and 10 scans were recorded and averaged.

In addition, fluorescence emission spectra of 0.9 μM holo-EE^47^, holo-ΔABC, denatured EE and denatured ΔABC were recorded. The enzymes were denatured by incubating 0.9 μM enzyme in 50 mM sodium phosphate, pH 7.4, containing 0.5% sodium dodecyl sulfate (SDS)^46^. After incubation at room temperature for several minutes, fluorescence emission spectra of the holo- and denatured enzymes were recorded as described above. Flavin composition of the denatured sample could be calculated using eqs (2) and (3):


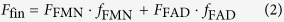



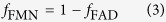


where *F*_fin_ is the final fluorescence emission after incubation of 0.9 μM holo enzyme with 0.5% SDS, and *F*_FMN_ and *F*_FAD_ are the fluorescence emissions of 0.9 μM FMN and 0.9 uM FAD, respectively. *f*_FMN_ and *f*_FAD_ are the fractions of FMN and FAD present in the sample.

### Mass spectrometry

To determine the FAD/FMN ratio in purified holoenzyme, the flavin cofactor was released from the enzyme by extraction with ethanol. 10 μM holo-EE and holo-ΔABC solutions were incubated with 60% ethanol for about 30 min. Subsequently, the solutions were centrifuged to remove aggregates. The solutions were passed through a 10 kDa cut-off Vivaspin 6 spinfilter (GE Healthcare) and the flow-throughs were collected. The solutions were freeze-dried and the obtained solids were dissolved in 120 μL pure water. Two microliter of these solutions were loaded onto a UPLC column (Discovery HS F5-3 2.1 mm I.D. × 150 mm, 3 μm particles, Sigma Aldrich). Separation was performed at 40 °C with a gradient from 100% H_2_O (with 0.1% formic acid) to 35% acetonitrile (with 0.1% formic acid) in 15 min at a flow rate of 250 μL/min. Use of the pentafluorophenylpropyl column enabled separation of FAD and FMN with only acetonitrile and water as solvents. This avoids contamination of the system as observed when tributylamine is used as counter ion^68^. Furthermore, the FAD and FMN peaks were without tailing in contrast to what has been observed with C18 chromatography^69^.

FMN and FAD were identified and quantified using a Shimadzu UPLC-triple quad mass spectrometer (LCMS-8040). The triple quad mass spectrometer was operating with 3 L/min nebulising gas flow and 15 L/min drying gas flow. The Dl temperature was set to 250 °C and the heat block temperature to 400 °C. Electrospray ionisation was used. The machine was calibrated with a reference set of FAD and FMN. The transition for FMN used for quantification was to the phosphate ion (97.0 m/z in negative mode), which was shown to be reproducible and quantitative. The fragmentation of FAD was to AMP (348.1 m/z in positive mode).

### Crystal growth, data collection and structure refinement for TtProDH ΔABC

TtProDH ΔABC crystals were obtained by the hanging-drop vapour-diffusion method at 292 K. Drops contained 0.5 μL of 2.2 mg mL^−1^ protein solution buffered with 50 mM Hepes, 500 mM sodium chloride, pH 8.0, and 0.5 μL of reservoir solution containing 20% (v/v) 2-propanol, 0.2 M calcium chloride dihydrate and 0.1 M sodium acetate, pH 4.6, and were equilibrated against 60 μL of reservoir solution. A mixture of 70% of reservoir solution and 30% of glycerol was used as cryoprotectant solution. A data set from one ΔABC monocrystal was collected in the beamline I02 of Diamond Light Source (Oxfordshire, UK) at a wavelength of 0.97949 Å and a temperature of 100 K. The data were processed and scaled using the XDS package^70^ and CCP4 software^71^. The crystal structure was solved by molecular replacement using the MOLREP program^72^ and the WT structure (PDB 2G37) as search model. Refinements were performed automatically by REFMAC 5 from CCP4^73^ and manually by COOT^74^. PROCHECK^75^ was used to assess and validate the final structure. TtProDH ΔABC diffracted up to 2.2 Å and belongs to the P6_2_ cubic space group. *V*_m_ value is 2.84 Å^3^/Da, with one protein molecule in the asymmetric unit and 56.68% of solvent content. Amino acid residues 280–296 and the AMP moiety of FAD, lacking observed electron density, are not included in the model. The Ramachandran plot shows that 97.08%, 2.5% and 0.42% of the amino acid residues are in most favoured, allowed and disallowed regions, respectively.

## Additional Information

**Accession codes:** The atomic coordinates and structure factors of TtProDH ΔABC have been deposited with the Protein Data Bank (www.rcsb.org) with the accession code 5M42.

**How to cite this article**: Huijbers, M. M. E. *et al*. Proline dehydrogenase from *Thermus thermophilus* does not discriminate between FAD and FMN as cofactor. *Sci. Rep.* **7**, 43880; doi: 10.1038/srep43880 (2017).

**Publisher's note:** Springer Nature remains neutral with regard to jurisdictional claims in published maps and institutional affiliations.

## Supplementary Material

Supplementary Figure S1

## Figures and Tables

**Figure 1:**
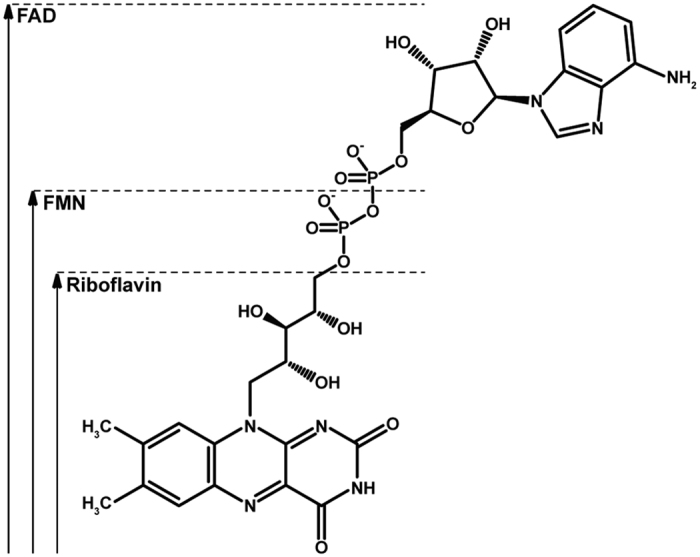
Chemical structure of riboflavin, FMN and FAD, with the redox-active isoalloxazine ring presented in the oxidised state.

**Table 1:** Specific activities and kinetic parameters of holo-EE, apo-EE and apo-EE reconstituted with FAD or FMN.

	Specific activity (U mg^−1^)	*k*_cat_ (s^−1^)	*K*_M_ (mM)	*k*_cat_/*K*_M_ (s^−1^ M^−1^)
Holo-EE*	4.74 ± 0.27	9.8 ± 0.5	67.6 ± 8.2	146
Apo-EE	0.84 ± 0.01	ND	ND	ND
Apo-EE + FAD	4.83 ± 0.07	7.2 ± 0.2	36.6 ± 3.2	198
Apo-EE + FMN	4.95 ± 0.10	7.9 ± 0.2	50.1 ± 3.4	158

Activity measurements were done at 25 °C, pH 7.4 with the proline: DCPIP oxidoreductase assay.

^*^As determined previously^47^.

**Table 2:** Data collection and refinement statistics for TtProDH ΔABC.

Data collection statistics	
Space group	P 6_2_
Cell dimensions a, b, c (Å)	131.92 131.92 36.58
Wavelength, Å	0.9794
Resolution, Å	65.96–2.2 (2.32–2.2)
Total no. of reflections	159591 (23667)
No. of unique reflections	18892 (2730)
Redundancy	8.4 (8.7)
Completeness, %	100 (100)
Average I/σ	11 (2.2)
R_merge_^a^	0.138 (0.897)
CC(1/2)	0.997 (0.714)
Refinement statistics	
Resolution range, Å	60–2.2
Protein non-hydrogen atoms	1977
Ligand non-hydrogen atoms	31
Solvent non-hydrogen atoms	65
R_work_ (%)	18.2
R_free_^b^ (%)	22.5
rmsd bond length, Å	0.019
rmsd bond angles, °	2.081
Average B-factor, Å^2^	37.67

Values in parentheses correspond to the highest resolution shell.

^a^R_merge_ = Σ(*I* − *I*_av_)*/*Σ*I*_av_, where the summation is over symmetry-equivalent reflections.

^b^R calculated for 5% of data excluded from the refinement.

## References

[b1] Joosten V. & van Berkel W. J. H. Flavoenzymes. Curr. Opin. Chem. Biol. 11, 195–202 (2007). 17275397 10.1016/j.cbpa.2007.01.010

[b2] Santos M. A., Jiménez A. & Revuelta J. L. Molecular characterization of *FMN1*, the structural gene for the monofunctional flavokinase of *Saccharomyces cerevisiae*. J. Biol. Chem. 275, 28618–28624 (2000). 10887197 10.1074/jbc.M004621200

[b3] Wu M., Repetto B., Glerum D. M. & Tzagoloff A. Cloning and characterization of FAD1, the structural gene for flavin adenine dinucleotide synthetase of *Saccharomyces cerevisiae*. Mol. Cell. Biol. 15, 264–271 (1995). 7799934 10.1128/mcb.15.1.264 PMC231949

[b4] Mack M., van Loon A. P. G. M. & Hohmann H. P. Regulation of riboflavin biosynthesis in *Bacillus subtilis* is affected by the activity of the flavokinase/flavin adenine dinucleotide synthetase encoded by *ribC*. J. Bacteriol. 180, 950–955 (1998). 9473052 10.1128/jb.180.4.950-955.1998 PMC106977

[b5] Manstein D. J. & Pai E. F. Purification and characterization of FAD synthetase from *Brevibacterium ammoniagenes*. J. Biol. Chem. 261, 16169–16173 (1986). 3023344

[b6] Serrano A., Ferreira P., Martínez-Júlvez M. & Medina M. The prokaryotic FAD synthetase family: a potential drug target. Curr. Pharm. Des. 19, 2637–2648 (2013). 23116401 10.2174/1381612811319140013

[b7] Macheroux P., Kappes B. & Ealick S. E. Flavogenomics–a genomic and structural view of flavin-dependent proteins. Febs J. 278, 2625–2634 (2011). 21635694 10.1111/j.1742-4658.2011.08202.x

[b8] de Gonzalo G., Smit C., Jin J., Minnaard A. J. & Fraaije M. W. Turning a riboflavin-binding protein into a self-sufficient monooxygenase by cofactor redesign. Chem. Commun. 47, 11050–11052 (2011). 10.1039/c1cc14039f 21901197

[b9] Hevel J. M., White K. A. & Marletta M. A. Purification of the inducible murine macrophage nitric oxide synthase. Identification as a flavoprotein. J. Biol. Chem. 266, 22789–22791 (1991). 1720773

[b10] Vermilion J. L. & Coon M. J. Identification of the high and low potential flavins of liver microsomal NADPH-cytochrome P-450 reductase. J. Biol. Chem. 253, 8812–8819 (1978). 31362

[b11] van Berkel W. J. H., Benen J. A. E. & Snoek M. C. On the FAD-induced dimerization of apo-lipoamide dehydrogenase from *Azotobacter vinelandii* and *Pseudomonas fluorescens*. Kinetics of reconstitution. Eur. J. Biochem. 197, 769–779 (1991). 2029906 10.1111/j.1432-1033.1991.tb15970.x

[b12] Müller F. & van Berkel W. J. H. A study on p-hydroxybenzoate hydroxylase from *Pseudomonas fluorescens*. A convenient method of preparation and some properties of the apoenzyme. Eur. J. Biochem. 128, 21–27 (1982). 6816593

[b13] Müller F. In Flavins and Flavoproteins: Methods and Protocols (eds Weber S. & Schleicher E.) 229–306 (Springer: New York, 2014).

[b14] Ghisla S. & Massey V. New flavins for old: artificial flavins as active site probes of flavoproteins. Biochem. J. 239, 1–12 (1986). 3541919 10.1042/bj2390001 PMC1147232

[b15] Krzek M., van Beek H. L., Permentier H. P., Bischoff R. & Fraaije M. W. Covalent immobilization of a flavoprotein monooxygenase via its flavin cofactor. Enzyme Microb. Technol. 82, 138–143 (2016). 26672460 10.1016/j.enzmictec.2015.09.006

[b16] Hefti M. H., Vervoort J. & van Berkel W. J. H. Deflavination and reconstitution of flavoproteins–Tackling fold and function. Eur. J. Biochem. 270, 4227–4242 (2003). 14622288 10.1046/j.1432-1033.2003.03802.x

[b17] Husain M. & Massey V. In Methods Enzymol., Volume 53 (eds Sidney F. & Lester P.) 429–437 (Academic Press, 1978).713848

[b18] Müller F. & van Berkel W. J. H. In Chemistry and biochemistry of flavoenzymes (ed Müller F.) 261–274 (CRC Press, Boca Raton 1991).

[b19] Van Berkel W. J. H. & Müller F. The elucidation of the microheterogeneity of highly purified *p*-hydroxybenzoate hydroxylase from *Pseudomonas fluorescens* by various biochemical techniques. Eur. J. Biochem. 167, 35–46 (1987). 3040401 10.1111/j.1432-1033.1987.tb13301.x

[b20] Mewies M., McIntire W. S. & Scrutton N. S. Covalent attachment of flavin adenine dinucleotide (FAD) and flavin mononucleotide (FMN) to enzymes: the current state of affairs. Protein Sci. 7, 7–20 (1998). 9514256 10.1002/pro.5560070102 PMC2143808

[b21] Heuts D. P. H. M., Scrutton N. S., McIntire W. S. & Fraaije M. W. What’s in a covalent bond? On the role and formation of covalently bound flavin cofactors. Febs J. 276, 3405–3427 (2009). 19438712 10.1111/j.1742-4658.2009.07053.x

[b22] Fraaije M. W., van den Heuvel R. H. H., van Berkel W. J. H. & Mattevi A. Covalent flavinylation is essential for efficient redox catalysis in vanillyl-alcohol oxidase. J. Biol. Chem. 274, 35514–35520 (1999). 10585424 10.1074/jbc.274.50.35514

[b23] Brandsch R. & Bichler V. Autoflavinylation of apo6-hydroxy-D-nicotine oxidase. J. Biol. Chem. 266, 19056–19062 (1991). 1918024

[b24] Fraaije M. W., van den Heuvel R. H. H., van Berkel W. J. H. & Mattevi A. Structural analysis of flavinylation in vanillyl-alcohol oxidase. J. Biol. Chem. 275, 38654–38658 (2000). 10984479 10.1074/jbc.M004753200

[b25] Tahallah N. . Cofactor-dependent assembly of the flavoenzyme vanillyl-alcohol oxidase. J. Biol. Chem. 277, 36425–36432 (2002). 12107187 10.1074/jbc.M205841200

[b26] Bandrin S. V., Rabinovich P. M. & Stepanov A. I. 3 linkage groups of genes of riboflavin biosynthesis in *Escherichia coli*. Genetika 19, 1419–1425 (1983). 6315532

[b27] Abbas C. A. & Sibirny A. A. Genetic control of biosynthesis and transport of riboflavin and flavin nucleotides and construction of robust biotechnological producers. Microbiol. Mol. Biol. Rev. 75, 321–360 (2011). 21646432 10.1128/MMBR.00030-10 PMC3122625

[b28] Hassan-Abdallah A., Bruckner R. C., Zhao G. & Jorns M. S. Biosynthesis of covalently bound flavin: isolation and *in vitro* flavinylation of the monomeric sarcosine oxidase apoprotein. Biochemistry 44, 6452–6462 (2005). 15850379 10.1021/bi047271x PMC1993914

[b29] Jin J. . Covalent flavinylation of vanillyl-alcohol oxidase is an autocatalytic process. Febs J. 275, 5191–5200 (2008). 18793324 10.1111/j.1742-4658.2008.06649.x

[b30] Adams E. & Frank L. Metabolism of proline and the hydroxyprolines. Annu. Rev. Biochem. 49, 1005–1061 (1980). 6250440 10.1146/annurev.bi.49.070180.005041

[b31] Phang J. M. The regulatory functions of proline and pyrroline-5-carboxylic acid. Curr. Top. Cell. Regul. 25, 91–132 (1985). 2410198 10.1016/b978-0-12-152825-6.50008-4

[b32] White T. A., Krishnan N., Becker D. F. & Tanner J. J. Structure and kinetics of monofunctional proline dehydrogenase from *Thermus thermophilus*. J. Biol. Chem. 282, 14316–14327 (2007). 17344208 10.1074/jbc.M700912200 PMC2708979

[b33] Tanner J. J. Structural biology of proline catabolism. Amino Acids 35, 719–730 (2008). 18369526 10.1007/s00726-008-0062-5 PMC2664619

[b34] Mitsubuchi H., Nakamura K., Matsumoto S. & Endo F. Inborn errors of proline metabolism. J. Nutr. 138, 2016S–2020S (2008). 18806117 10.1093/jn/138.10.2016S

[b35] Phang J. M., Hu C. A. & Valle D. In Metabolic and Molecular Bases of Inherited Disease (eds Scriver C. R., Beaudet A. L., Sly W. S. & Valle D.) 1821–1838 (McGraw-Hill, 2001).

[b36] Hu C. A. . Functional genomics and SNP analysis of human genes encoding proline metabolic enzymes. Amino Acids 35, 655–664 (2008). 18506409 10.1007/s00726-008-0107-9 PMC2707926

[b37] Bender H. U. . Functional consequences of PRODH missense mutations. Am. J. Hum. Genet. 76, 409–420 (2005). 15662599 10.1086/428142 PMC1196393

[b38] Jacquet H. . Hyperprolinemia is a risk factor for schizoaffective disorder. Mol. Psychiatry 10, 479–485 (2004). 10.1038/sj.mp.4001597 15494707

[b39] Jacquet H. . PRODH mutations and hyperprolinemia in a subset of schizophrenic patients. Hum. Mol. Genet. 11, 2243–2249 (2002). 12217952 10.1093/hmg/11.19.2243

[b40] Willis A., Bender H. U., Steel G. & Valle D. PRODH variants and risk for schizophrenia. Amino Acids 35, 673–679 (2008). 18528746 10.1007/s00726-008-0111-0

[b41] Donald S. P. . Proline oxidase, encoded by p53-induced gene-6, catalyzes the generation of proline-dependent reactive oxygen species. Cancer Res. 61, 1810–1815 (2001). 11280728

[b42] Polyak K., Xia Y., Zweier J. L., Kinzler K. W. & Vogelstein B. A model for p53-induced apoptosis. Nature 389, 300–305 (1997). 9305847 10.1038/38525

[b43] Liu W. & Phang J. M. Proline dehydrogenase (oxidase) in cancer. Biofactors 38, 398–406 (2012). 22886911 10.1002/biof.1036 PMC7479541

[b44] Lee Y. H., Nadaraia S., Gu D., Becker D. F. & Tanner J. J. Structure of the proline dehydrogenase domain of the multifunctional PutA flavoprotein. Nat. Struct. Biol. 10, 109–114 (2003). 12514740 10.1038/nsb885 PMC3727246

[b45] Guenther B. D. . The structure and properties of methylenetetrahydrofolate reductase from *Escherichia coli* suggest how folate ameliorates human hyperhomocysteinemia. Nat. Struct. Biol. 6, 359–365 (1999). 10201405 10.1038/7594

[b46] Huijbers M. M. E. & van Berkel W. J. H. High yields of active *Thermus thermophilus* proline dehydrogenase are obtained using maltose-binding protein as a solubility tag. Biotechnol. J. 10, 395–403 (2015). 25545499 10.1002/biot.201400229

[b47] Huijbers M. M. E. & van Berkel W. J. H. A more polar N-terminal helix releases *Thermus thermophilus* proline dehydrogenase from self-association. J. Mol. Catal. Enzym. B 134, 340–346 (2016).

[b48] Weber G. Fluorescence of riboflavin and flavin-adenine dinucleotide. Biochem. J. 47, 114–121 (1950). 14791317 10.1042/bj0470114 PMC1275171

[b49] Visser A. J. W. G. Kinetics of stacking interactions in flavin adenine dinucleotide from time-resolved flavin fluorescence. Photochem. Photobiol. 40, 703–706 (1984). 6522458 10.1111/j.1751-1097.1984.tb04640.x

[b50] Scarpulla R. C. & Soffer R. L. Membrane-bound proline dehydrogenase from *Escherichia coli*. Solubilization, purification and characterization. J. Biol. Chem. 253, 5997–6001 (1978). 355248

[b51] Menzel R. & Roth J. Enzymatic properties of the purified *putA* protein from *Salmonella typhimurium*. J. Biol. Chem. 256, 9762–9766 (1981). 6270101

[b52] Menzel R. & Roth J. Purification of the *putA* gene product - A bifunctional membrane-bound protein from *Salmonella typhimurium* responsible for the two-step oxidation of proline to glutamate. J. Biol. Chem. 256, 9755–9761 (1981). 6270100

[b53] Bollen Y. J. M., Nabuurs S. M., van Berkel W. J. H. & van Mierlo C. P. M. Last in, first out: the role of cofactor binding in flavodoxin folding J. Biol. Chem. 280, 7836–7844 (2005). 15632150 10.1074/jbc.M412871200

[b54] Singh H., Arentson B. W., Becker D. F. & Tanner J. J. Structures of the PutA peripheral membrane flavoenzyme reveal a dynamic substrate-channeling tunnel and the quinone-binding site. Proc. Natl. Acad. Sci. USA 111, 3389–3394 (2014). 24550478 10.1073/pnas.1321621111 PMC3948300

[b55] Srivastava D. . Crystal structure of the bifunctional proline utilization A flavoenzyme from *Bradyrhizobium japonicum*. Proc. Natl. Acad. Sci. USA 107, 2878–2883 (2010). 20133651 10.1073/pnas.0906101107 PMC2840367

[b56] Luo M., Arentson B. W., Srivastava D., Becker D. F. & Tanner J. J. Crystal structures and kinetics of monofunctional proline dehydrogenase provide insight into substrate recognition and conformational changes associated with flavin reduction and product release. Biochemistry 51, 10099–10108 (2012). 23151026 10.1021/bi301312f PMC3525754

[b57] Zhang M. . Structures of the *Escherichia coli* PutA proline dehydrogenase domain in complex with competitive inhibitors. Biochemistry 43, 12539–12548 (2004). 15449943 10.1021/bi048737e PMC3727243

[b58] van der Laan J. M. . The coenzyme analog adenosine 5-diphosphoribose displaces FAD in the active site of p-hydroxybenzoate hydroxylase. An x-ray crystallographic investigation. Biochemistry 28, 7199–7205 (1989). 2819062 10.1021/bi00444a011

[b59] Sato K., Nishina Y. & Shiga K. The binding of adenine nucleotides to apo-electron-transferring flavoprotein. J. Biochem. 112, 804–810 (1992). 1295890 10.1093/oxfordjournals.jbchem.a123980

[b60] Tedeschi G., Negri A., Ceciliani F., Mattevi A. & Ronchi S. Structural characterization of L-aspartate oxidase and identification of an interdomain loop by limited proteolysis. Eur. J. Biochem. 260, 896–903 (1999). 10103021 10.1046/j.1432-1327.1999.00234.x

[b61] Lindqvist Y. Refined structure of spinach glycolate oxidase at 2 Å resolution. J. Mol. Biol. 209, 151–166 (1989). 2681790 10.1016/0022-2836(89)90178-2

[b62] Xia Z. X. & Mathews F. S. Molecular structure of flavocytochrome *b*_2_ at 2.4 Å resolution. J. Mol. Biol. 212, 837–863 (1990). 2329585 10.1016/0022-2836(90)90240-M

[b63] Fox K. M. & Karplus P. A. Old yellow enzyme at 2 Å resolution: overall structure, ligand binding, and comparison with related flavoproteins. Structure 2, 1089–1105 (1994). 7881908

[b64] Lim L. W. . Three-dimensional structure of the iron-sulfur flavoprotein trimethylamine dehydrogenase at 2.4 Å resolution. J. Biol. Chem. 261, 15140–15146 (1986). 3771568

[b65] Rowland P., Nielsen F. S., Jensen K. F. & Larsen S. The crystal structure of the flavin containing enzyme dihydroorotate dehydrogenase A from *Lactococcus lactis*. Structure 5, 239–252 (1997). 9032071 10.1016/s0969-2126(97)00182-2

[b66] Park H. J. . Purification and characterization of a NADH oxidase from the thermophile *Thermus thermophilus* HB8. Eur. J. Biochem. 205, 881–885 (1992). 1577005 10.1111/j.1432-1033.1992.tb16853.x

[b67] Hecht H. J., Erdmann H., Park H. J., Sprinzl M. & Schmid R. D. Crystal structure of NADH oxidase from *Thermus thermophilus*. Nat. Struct. Biol. 2, 1109–1114 (1995). 8846223 10.1038/nsb1295-1109

[b68] Buescher J. M., Moco S., Sauer U. & Zamboni N. Ultrahigh performance liquid chromatography−tandem mass spectrometry method for fast and robust quantification of anionic and aromatic metabolites. Anal. Chem. 82, 4403–4412 (2010). 20433152 10.1021/ac100101d

[b69] Mialoundama A. S. . Characterization of plant carotenoid cyclases as members of the flavoprotein family functioning with no net redox change. Plant Physiol. 153, 970–979 (2010). 20460582 10.1104/pp.110.155440 PMC2899934

[b70] Kabsch W. X. D. S. Acta Crystallogr. D Biol. Crystallogr. 66, 125–132 (2010). 20124692 10.1107/S0907444909047337 PMC2815665

[b71] Collaborative Computational Project, N. The *CCP*4 suite: programs for protein crystallography. Acta Crystallogr. D Biol. Crystallogr. 50, 760–763 (1994). 15299374 10.1107/S0907444994003112

[b72] Vagin A. A. New translation and packing functions. Newsletter on protein crystallography, Daresbury Laboratory 24, 117–121 (1989).

[b73] Murshudov G. N., Vagin A. A. & Dodson E. J. Refinement of macromolecular structures by the maximum-likelihood method. Acta Crystallogr. D Biol. Crystallogr. 53, 240–255 (1997). 15299926 10.1107/S0907444996012255

[b74] Emsley P., Lohkamp B., Scott W. G. & Cowtan K. Features and development of *Coot*. Acta Crystallogr. D Biol. Crystallogr. 66, 486–501 (2010). 20383002 10.1107/S0907444910007493 PMC2852313

[b75] Laskowski R. A., MacArthur M. W., Moss D. S. & Thornton J. M. *PROCHECK*: a program to check the stereochemical quality of protein structures. J. Appl. Cryst. 26, 283–291 (1993).

