# Supplementary material for: Proline dehydrogenase from Thermus thermophilus does not discriminate between FAD and FMN as cofactor
Source: Sci Rep. 2017 Mar 3;7:43880. doi: 10.1038/srep43880 (PMC5335563; doi:10.1038/srep43880)
Supplement: Supplementary Figure S1 [file srep43880-s1.pdf]

Proline dehydrogenase from *Thermus thermophilus* does not discriminate between FAD and FMN as cofactor

Mieke M.E. Huijbers, Marta Martínez-Júlvez, Adrie H. Westphal, Estela Delgado-Arciniega, Milagros Medina and Willem J.H. van Berkel

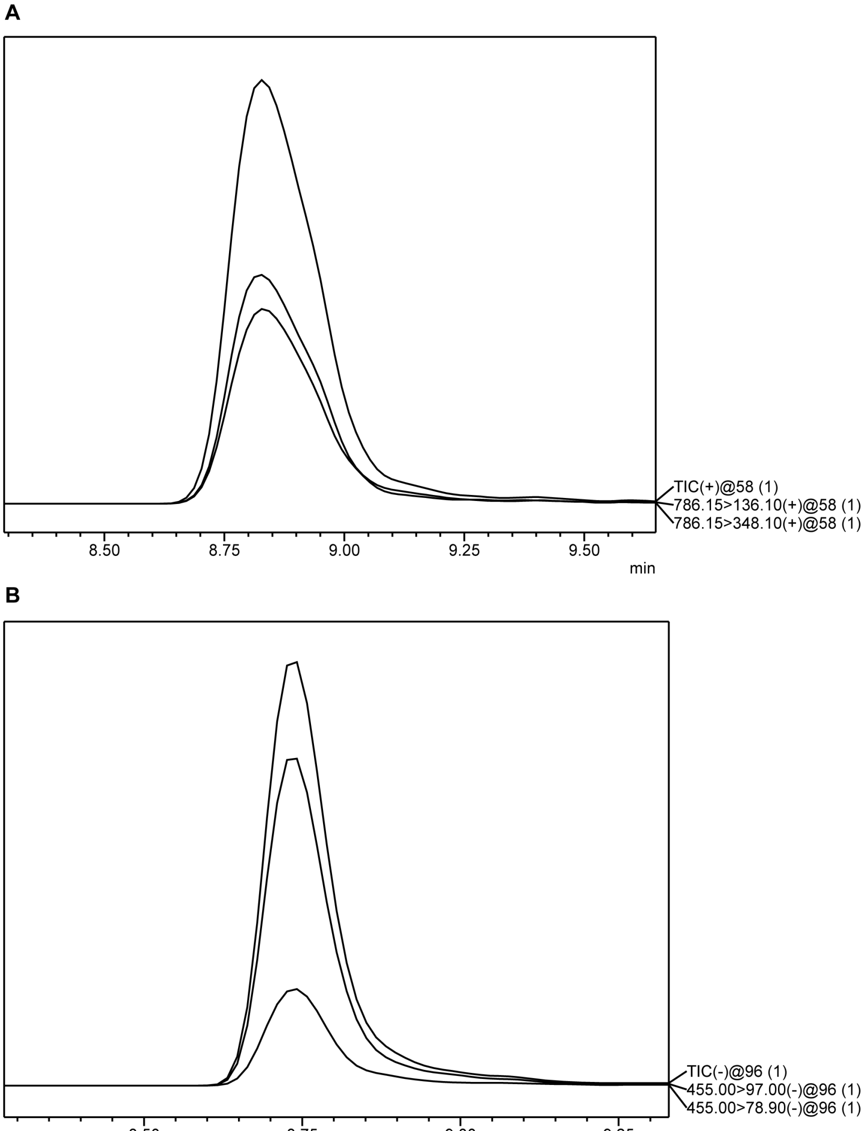

Figure S1.
Analysis of the flavin extracted from TtProDH ΔABC using a Shimadzu UPLC-triple quad mass spectrometer (LC/MS-8040).
(A) Detection of FAD in positive mode for the transition of 786.15 to 136.10 m/z and from 786.15 to 348.10 m/z at 8.85 min.
(B) Detection of FMN in negative mode for the transition of 455.00 to 97.00 m/z and from 455.00 to 78.90 m/z at 8.73 min.
